# Phylogenetic and AlphaFold predicted structure analyses provide insights for A1 aspartic protease family classification in *Arabidopsis*

**DOI:** 10.3389/fpls.2023.1072168

**Published:** 2023-02-03

**Authors:** Yanling Duan, Hao Tang, Xiaobo Yu

**Affiliations:** Bamboo Diseases and Pest control and Resources Development Key Laboratory of Sichuan Province, College of Life Science, Leshan Normal University, Leshan, China

**Keywords:** aspartic protease, AlphaFold, phylogentic analysis, cysteine residues, *Arabidopsis thaliana*

## Abstract

Aspartic proteases are widely distributed in animals, plants, fungi and other organisms. In land plants, A1 aspartic protease family members have been implicated to play important and varied roles in growth, development and defense. Thus a robust classification of this family is important for understanding their gene function and evolution. However, current A1 family members in *Arabidopsis* are less well classified and need to be re-evaluated. In this paper, 70 A1 aspartic proteases in *Arabidopsis* are divided into four groups (group I-IV) based on phylogenetic and gene structure analyses of 1200 A1 aspartic proteases which are obtained from 12 Embryophyta species. Group I-III members are further classified into 2, 4 and 7 subgroups based on the AlphaFold predicted structures. Furthermore, unique insights of A1 aspartic proteases have been unraveled by AlphaFold predicted structures. For example, subgroup II-C members have a unique II-C specific motif in the C-extend domain, and subgroup IV is a Spermatophyta conserved group without canonical DTGS/DSGT active sites. These results prove that AlphaFold combining phylogenetic analysis is a promising solution for complex gene family classification.

## Introduction

Proteases regulate various biological processes including protein synthesis and maturation, activity modification, degradation and turnover. Depending on their catalytic mechanisms, these proteases are primarily classified into cysteine, metallo-, serine, threonine and aspartic protease family (Beers et al., 2004). The latter protease family is known as acid protease family because they are most active at acid pH. In the protease MEROPS database (http://merops.sanger.ac.uk), aspartic proteases are divided into A1, A2, A3, A5, A8, A9, A11, A22, A24, A25, A26, A28, A31, A32, A36, A37 family (Rawlings et al., 2010). The majority of animal and plant aspartic proteases belong to the A1 family, and one of the best characterized protease is pepsin A, which is the main digestive protease in the animal stomach (Fruton, 2002). A1 aspartic protease is thus called pepsin-like aspartic protease.

Compared with animals, A1 family in plants is more complex, which is reflected not only by the gene number but also by their structural variations. Thus the classification of A1 family in plants is challenging, and several papers have attempted to identify and classify this family in the model species *Arabidopsis thaliana* (Beers et al., 2004; Faro and Gal, 2005; Takahashi et al., 2008). We redraw these classifications in **Figures S1**–**S3** to facilitate comparison. 59 A1 members were identified and classified into A1-1 to A1-5 group in 2004 (**Figure S1**) (Beers et al., 2004). 51 A1 members were identified and classified into typical type, nucellin-like type and atypical type in 2005 (**Figure S2**) (Faro and Gal, 2005). Then 63 A1 members were identified and classified into nepenthesin-like aspartic proteases (NAPs) subfamily and pepsin-like proteases (PAPs) subfamily in 2008 (**Figure S3**) (Takahashi et al., 2008). Taken together, these classification systems are somewhat arbitrary and ambiguous. Moreover, the classification basis is also ambiguous. For example, nucellin-like APs were distinguished by two sequence features, which are (1) the first active site (acidic-hydrophobic-DTG-serine-acidic) and (2) two conserved sequences (QCDYE and GCGYDQ) located in between the first DTG and the GILGL sequence (Faro and Gal, 2005). However, these features should be carefully considered. Firstly, though the first active site of nucellin-like APs are D (acidic)-I/L (hydrophobic)-DTGS-acidic, such active site motif can also be found in atypical APs such as AT2G28030, AT2G28220 and AT2G28225 (EIDTGSD), indicating that this sequence cannot be treated as the unique feature of nucellin-like APs. Secondly, two cysteine residues contained QCDYE and GCGYDQ motifs are not even conserved in nucellin itself (RCHDE and GCGYKQ).Taken together, these classification systems should be re-evaluated to better understand their biological functions and evolution.

Well-investigated biological and biochemical properties, robust phylogenetic relationships and structural information are essential factors for a good classification system. Major advances have been made in our understanding of the biological and biochemical properties of *Arabidopsis* APs during the past two decades (Xia et al., 2004; Ge et al., 2005; Simões et al., 2007; Phan et al., 2011; Paparelli et al., 2012; Yao et al., 2012; Breitenbach et al., 2014; Li et al., 2016; Gao et al., 2017; Soares et al., 2019a; Soares et al., 2019b; Wang et al., 2019; Yu et al., 2021). In contrast to the progress of biological functions, the knowledge of plant APs’ structural information is still largely missing and only two A1 aspartic proteases (phytepsin and cardosin A) have been crystallized in plants (Frazão et al., 1999; Kervinen et al., 1999). The recently developed AlphaFold, which can predict highly accurate protein structures, has greatly expanded our understanding of structural biology (Jumper et al., 2021), thus offering us an opportunity to explore the structural information of A1 family members.

In this paper, AlphaFold predicted three-dimensional structural models combined with traditional phylogenetic analysis are used to reclassify and provide new insights for the A1 aspartic protease family in *Arabidopsis.*

## Methods

### Identification of A1 aspartic protease family genes

Combined strategies were used to identify A1 aspartic protease genes in *Arabidopsis thaliana*. The keyword “aspartic protease” was used as a query to search in the TAIR database (https://www.arabidopsis.org/), Uniprot database (https://www.uniprot.org/) and Ensemblplants database (http://plants.ensembl.org/) to obtain possible AP sequences. The Hidden Markov Model (HMM) profile of ASP domain (PF00026) was used as a query to search against *Arabidopsis* genome, and the obtained protein sequences were used to create a custom made ASP HMM profile by HMMER 3.1b2, and this custom made ASP HMM profile was used as a query for a another round search. These obtained sequences were manually compared with the “keyword” obtained sequences of three databases, then pseudogenes and erroneously annotated genes were manually eliminated based on AlphaFold predicted structures, and finally A1 aspartic protease genes were identified.

To identify A1 aspartic protease genes in *Marchantia polymorpha*, *Amborella trichopoda*, *Oryza sativa ssp*. *Japonica*, *Glycine max*, *Citrus clementina*, *Lactuca sativa*, *Solanum tuberosum*, *Medicago truncatula*, *Brassica napus* and *Capsella rubella*, protein sequences of A1 aspartic protease genes in *Arabidopsis thaliana* were used as queries to obtain possible AP genes by BLAST program. These obtained sequences were further verified by the custom made ASP HMM profile.

### Multiple sequence alignment and phylogenetic analysis

Protease domains of all AP genes were extracted based on the custom made ASP HMM profile. Multiple sequence alignments were aligned through Multiple Alignment using Fast Fourier Transform (MAFFT). T-coffee’s TCS program was used to determine the quality of the MSA, and the resulting MSA was further trimmed by BMGE (entropy between 0.7 and 0.9). The trimmed MSA was used to construct phylogenetic tree by RAXML with Maximum Likelihood (ML) method and a 1000 times bootstrap analysis.

### Gene structure analysis

Average intron and exon numbers of group I-IV in all 12 selected species were analyzed and computed by a self written Perl script based on the gff files of all identified genes.

### AlphaFold predicted structures analysis

All analyzed pdb files are downloaded from AlphaFold Protein Structure Database (https://alphafold.ebi.ac.uk/), and these structure models were visualized by PyMOL (Yuan et al., 2017). The pdb file of human pepsin with inhibitor pepsatin (1pso) was downloaded from PDB database (https://www.rcsb.org/).

## Results and discussion

### A1 aspartic protease content in *Arabidopsis* genome

Though A1 aspartic protease family members have been identified and described by different investigators in last two decades, the gene number of A1 family ranges from 51 to 69 (Beers et al., 2004; Faro and Gal, 2005; Takahashi et al., 2008; Wang et al., 2019; Yu et al., 2021). To obtain as many A1 members as possible in *Arabidopsis*, the HMM profile of ASP domain (PF00026), which has been used to identify A1 family genes in rice (Chen et al., 2009), grape (Guo et al., 2013), poplar(Cao et al., 2019), wheat (Yang and Feng, 2020), moso bamboo (Wang et al., 2021) and potato (Norero et al., 2022), was used as a query to identify AP genes in the first round search. Then only 64 genes were identified (**Table S1**), which is obviously underestimated since reported aspartic proteases such as SAP1(AT1G03220) and SAP2(AT1G03230) cannot be not identified (Wang et al., 2019). Therefore the ASP HMM profile (PF00026) should be corrected to obtain all possible AP genes. Thus we then used the identified 64 sequences to create a custom made ASP HMM profile, which was used as a query to perform a new round search. A total of 70 genes including SAP1 and SAP2 are identified (**Table S2**). We also used “aspartic protease” as the key word to search several databases such as TAIR, Uniprot and Ensemblplants databases, 73 gene models are found but 3 genes were eliminated (**Table S3**, **Figure S4**). Finally, 70 A1 aspartic protease genes were identified in the *Arabidopsis thaliana* genome.

### Phylogenetic and gene structure analysis provide insights for A1 aspartic protease family classification

**Figure 1:**
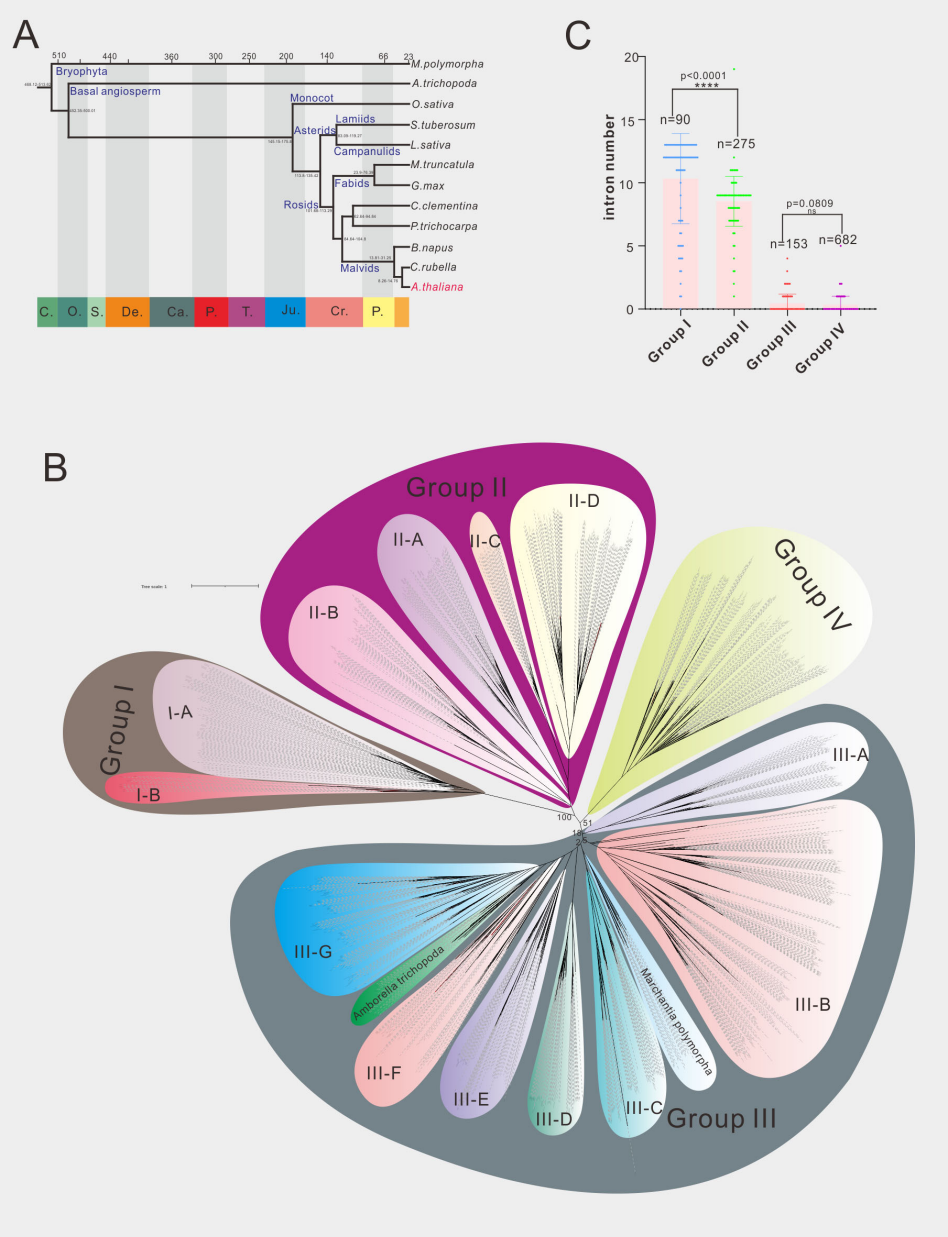
Phylogenetic analysis of A1 aspartic protease family in 12 selected species. **(A)** The evolution relationship of 12 selected species. The top of tree showed the absolute age, unit: million years, the bottom of the tree showed the geologic time, C, Cambrian; O, Ordovician; S, Silurian; De, Devonian; Ca, Carboniferous; P, Permian; T, Triassic; Ju, Jurassic; Cr, Cretaceous; P, Paleogene (from left to right). **(B)** Phylogenetic tree of A1 family members in 12 selected species, maximum likelihood method (ML) was applied with a 1000 bootstrap analysis, groups and subgroups are labeled. **(C)** Average intron numbers of group I-IV, n presents gene numbers in each group.

Phylogenetic analysis is the most commonly used method for gene family classification. We thus constructed a phylogenetic tree of 70 A1 aspartic protease family in *Arabidopsis* (**Figure S5**). In order to facilitate comparison, we redraw three current existed classification systems of A1 family in *Arabidopsis* (**Figures S1**–**S3**). Both the gene numbers and classification were different from each other. Here we identified 70 A1 aspartic proteases and classified them into four groups (Group I-IV) (**Figures 1B**; **S5**). To provide a more robust classification system, different species covering dicots, monocots, basal angiosperm and bryophytes were analyzed. In detail, nine dicot species, one monocot species *Oryaza Sativa*, basal angiosperm *Amborella trichopoda* and bryophytes *Marchantia polymorpha* were selected for further analysis (**Figure 1A**; **Table S4**). In dicots, two asterids species (*Solanum tuberosum* represents lamiids, *Lactuca sativa* represents campanulids) and seven rosids species (*Glycine max*, *Medicago truncatula* and *Populus trichocarpa* represent fabids, *Arabidopsis thaliana*, *Brassica napus* and *Capsella rubella* and *Citrus clementina* represent Malvids) were included. A total of 1200 A1 aspartic protease genes were identified and were used to construct the phylogenetic tree (**Figure 1B**; **Supplemental Datafile 1**). The result showed that four groups are clearly distinguished, 90 genes were classified into group I, 275 genes are classified into group II, 682 genes were classified into group III, and 153 genes were classified into group IV (**Figure 1C**). To explore other features to distinguish these groups, we then investigated their genetic structures. The results showed that group I members have an average of 10.33 introns, group II members have an average of 8.53 introns, group III members have an average of 0.45 intron(s), and group IV members have an average of 0.33 intron(s) (**Figure 1C**), indicating group I and group II members have much more introns than group III and group IV members. Almost all 12 selected species have similar genetic structures in these four groups (**Tables S5**, **S6**), indicating exon-intron organization is a conserved feature of A1 aspartic protease family and can be used to distinguish these groups not only in *Arabidopsis* but also in other land plants.

### AlphaFold predicted three-dimensional structures provide insights for A1 aspartic protease classification

**Figure 2:**
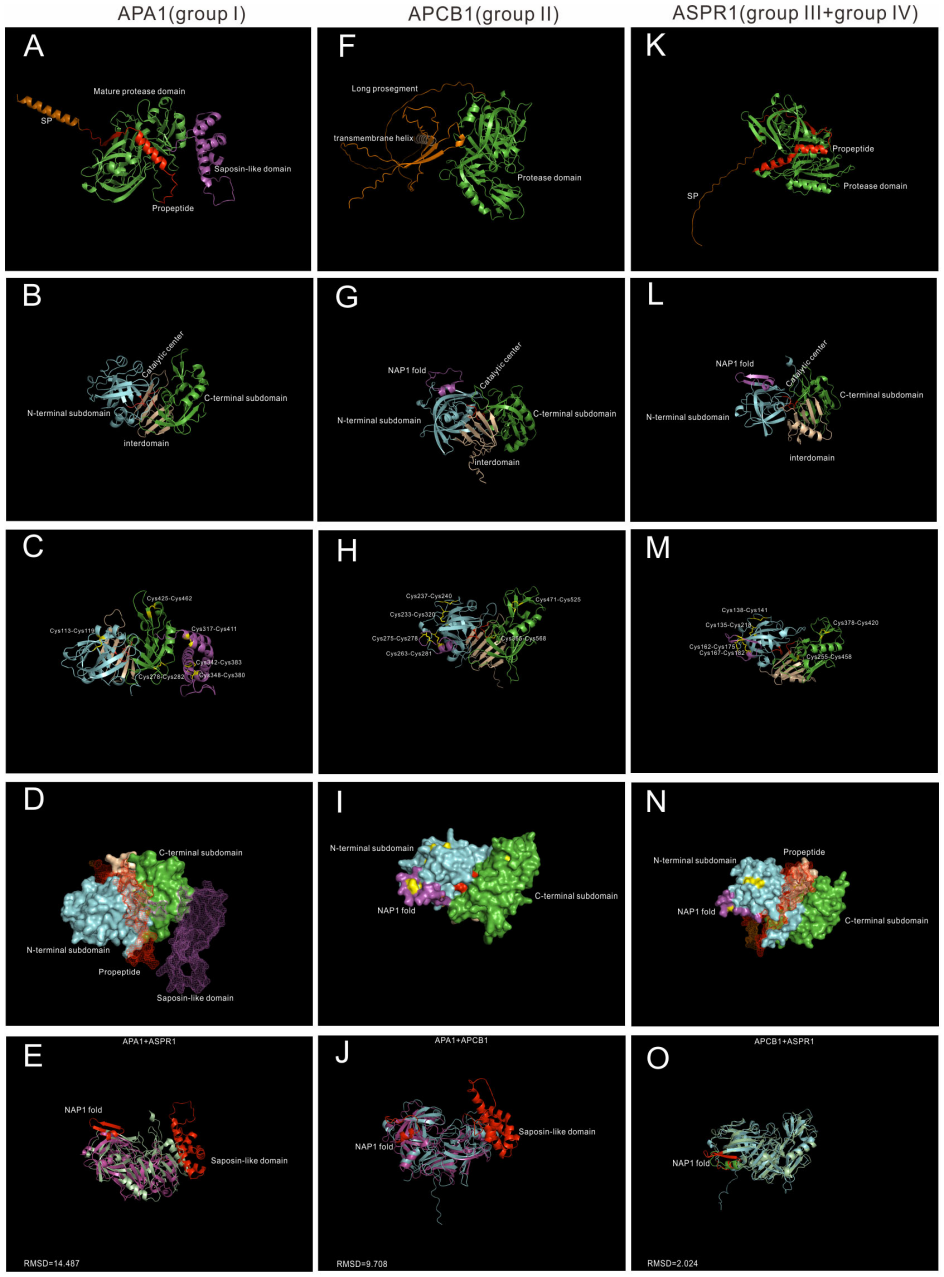
AlphaFold predicted structure models of Group I-IV. **(A–D)** Group I typical structure (APA1). **(A)** overall structure of APA1. SP,signal peptide, is colored by orange; propeptide is colored by red, saposin-like domain is colored by purple. **(B)** Proposed mature protease domain of APA1. N-terminal subdomain is colored by cyan, C-terminal subdomain is colored by green, interdomain beta-sheet is colored by wheat, and active sites are colored by red. **(C)** Disulfide bonds of APA1. **(D)** Surface and mesh of APA. The proposed mature protease structure is presented by surface, propeptide and saposin-like domain is presented by mesh. **(F–I)** Group II typical structure (APCB1). **(F)** Overall structure of APCB1.Long prosegment is colored by orange and transmembrane helix is colored by wheat. **(B)** Proposed protease domain of APCB1,NAP1 fold is clored by purple. **(H)** Disulfide bonds of APCB1. **(I)** surface of APCB1. **(K–N)** Group III typical structure (ASPR1). **(K)** Overall structure of APSR1. **(L)** proposed mature protease structure of ASPR1. **(M)** Surface of ASPR1. **(N)** Disulfide bonds of ASPR1. **(E–O)** Superposition analysis of APA1, APCB1 and ASPR1, RMSD value are presented.

To explore the underlying structural mechanisms of group I-IV, recent developed AlphaFold, which can predict protein structures with highly accuracy (Jumper et al., 2021), was applied to investigate these proteases at the structure levels. Here three proteases, whose biological functions and/or chemical properties are well studied, are used for structural analysis. APA1 (AT1G11910) represents group I, APCB1 (AT1G04950) represents group II, and ASPR1(AT2G03200) represents group III and group IV (**Figure 2**).

APA1 is a vacuolar aspartic protease, which is predominantly expressed in seeds (Chen et al., 2002). It has a N-terminal signal peptide(1-24aa), a propeptide (25-64aa), a saposin-like domain (312-417aa) and a pepsin-like aspartic protease domain (**Figure 2A**). The mature protease domain is composed of N-terminal subdomain and C-terminal domain, which are anchored *via* five stranded interdomain β-sheet (**Figure 2B**). There are six disulfide bridges formed by 12 cysteine residue. The mature protease domain is stabilized by three disulfide bridges (Cys113-Cys119, Cys278-Cys282 and Cys425-Cys462), and the saposin-like domain is stabilized by the other three disulfide bridges (Cys317-Cys411, Cys342-Cys383 and Cys348-Cys380) (**Figure 2C**). APA1 is likely synthesized as an inactive proenzyme because the catalytic center is covered by a propetide (**Figure 2D**).The saposin-like domain is related with its vacuolar location (**Figure 2D**) (Chen et al., 2002).

APCB1 is a plasma membrane located aspartic protease, which plays vital roles in autophagy and fungal resistance (Li et al., 2016). It has a very long prosegment sequence and a nucellin-like protease domain. And there exists a transmembrane helix in the prosegment, which may be responsible its membrane location (**Figure 2F**). The N-terminal subdomain and C-terminal domain are anchored *via* six rather than five stranded interdomain β-sheet. There is a nepenthesin 1-type’ aspartyl protease (NAP1) fold similar insert sequence (Athauda et al., 2004) in the N-terminal subdomain (**Figure 2G**). The protease domain of APCB1 is stabilized by six disulfide bonds formed by 12 cysteine residues, four bonds exist in N-terminal subdomain, one bond exists in interdomain(Cys365-Cys568) and the other bond in C-terminal subdomain(Cys471-Cys525). In the N-terminal subdomain, two disulfide bonds stabilize the NAP1 fold (Cys263-Cys281 and Cys275-Cys278), the other two bonds stabilize the N-terminal subdomain (Cys233-Cys320 and Cys237-Cys240) (**Figure 2H**). The NAP1 fold forms a surface loop and is proposed to be a molecular gate (Hodder et al., 2015).Consistently, the catalytic cleft of APCB1 is not sufficiently open (**Figure 2I**) and its activity is regulated by its binding proteins BAGP1 (Li et al., 2016).

ASPR1 is a secreted aspartic protease, which plays roles in lateral root development (Soares et al., 2019a). It is composed of a signal peptide, a propeptide and a protease domain(**Figure 2K**). The overall structure of ASPR1 is similar to APCB1,but there is an extra alpha helix in the interdomain with unknown functions (**Figure 2L**). Similarly, the protease domain of ASPR1 is also stabilized by six disulfide bridges formed by 12 cysteine residues. In the N-terminal subdomain, the NAP1 fold is nestled by two disulfide linkages (Cys162-Cys175 and Cys167-Cys182), the other two linkages in the N-subdomain are Cys135-Cys218 and Cys138-Cys141 (**Figure 2M**). The catalytic cleft of ASPR1 is covered by a propetide (**Figure 2N**) (Soares et al., 2019a).

Taken together, APCB1 and ASPR1 are structural similar (RMSD = 2.024) (**Figure 2O**), but both proteases are different from APA1 (APA1+APCB1,RMSD = 14.487;APA1+ASPR1,RMSD = 9.708) (**Figures 2E–J**). The most notable difference between APCB1 and ASPR1 is their subcellular locations. In fact, many group II members are membrane located such as APCB1, A36 and A39, but many group III members are secreted proteases such as ASPR1,CDR1,ECS1 and ECS2.

### New classification of A1 aspartic protease family

Based on the results of phylogentic analysis, gene structure analysis and AlphaFold predicted structures, A1 aspartic protease family, these groups are further classified into several subgroups, the overall distribution of these subgroups are displayed in **Figure S6B**. In the followings, these subgroups are described and analyzed in detail.

### Group I

**Figure 3:**
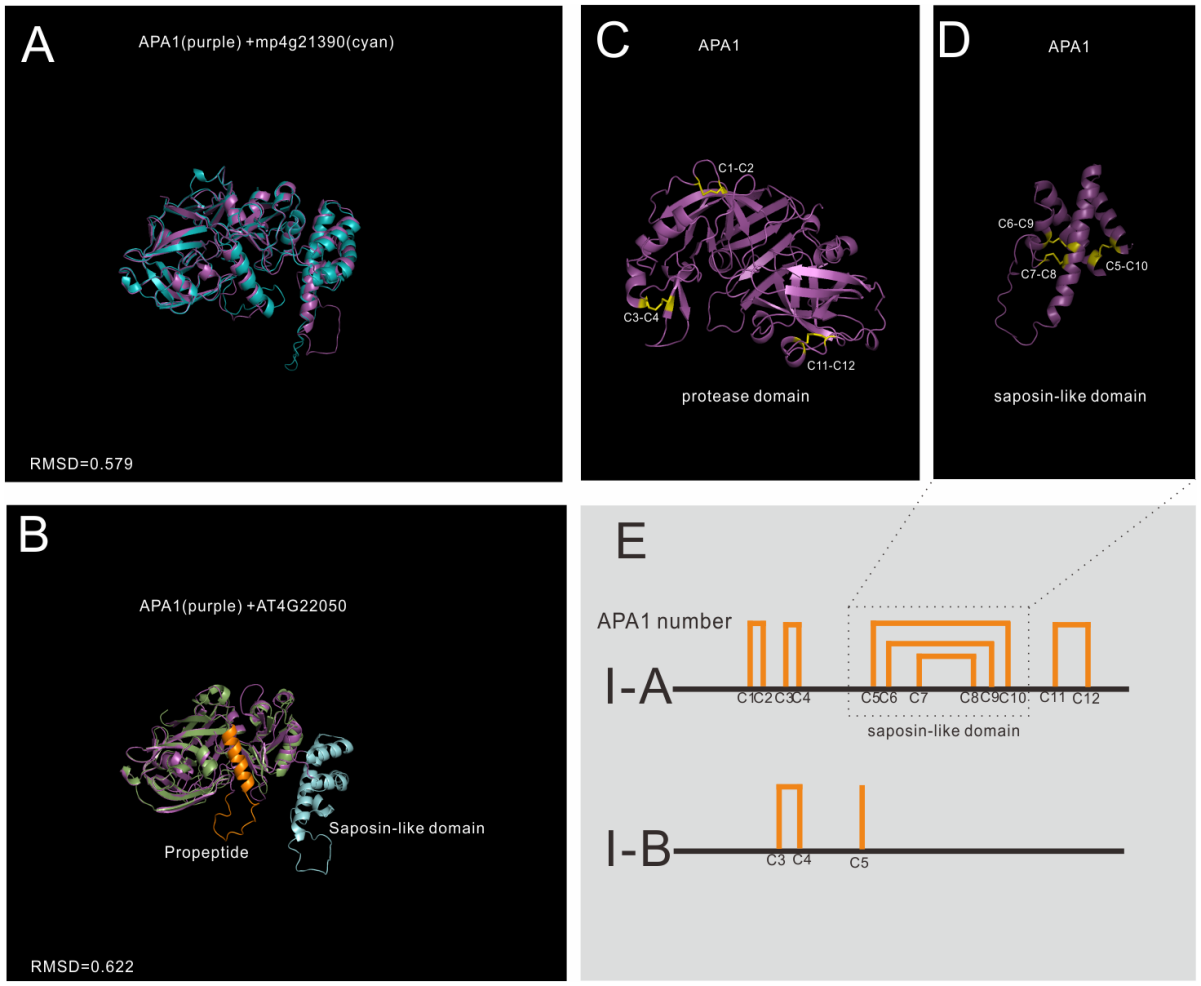
AlphaFold predicted structure models of group I (subgroup I-A and subgroup II-B) **(A)** Structure alignment of APA1 and its Marchantia polymorpha mp4g21390; **(B)** Structure alignment of subgroup I-A (APA1) and subgroup I-B (AT4G22050); **(C)** The disulfide bonds of mature APA1 protease domain; **(D)** The disulfide bonds of saposin-like domain; **(E)** Disulfide bond topology of subgroup I-A and I-B.

Group I has the least members in A1 family, and 5 genes in *Arabidopsis* are grouped into group I, which is previously identified as typical type (Faro and Gal, 2005). The structures of mature enzyme are similar, which are stabilized by three conserved disulfide bonds. In BMGE trimmed MSA, the saposin-like domain was trimmed and the MSA result showed that group I members have a typical-like cysteine residues (**Supplemental Datafile 2**). In group I, 3 genes have a saposin-like domain, and the other two genes lack this domain, thus group I was further classified into subgroup I-A and I-B (**Figure 3**).

Subgroup I-A is distributed in Embryophyta (**Figure 3A** show the alignment between APA1 and mp4g21390, RMSD=0.579), and there are three members in *Arabidopsis*, which are AT1G11910 (APA1), AT1G62290 (APA2) and AT4G04460 (APA3). The protease domain and saposin-like domain are stabilized by six disulfide bonds (12 cysteine number termed by C1-C12). The protease domain is stabilized by three disulfide bonds, two bonds in N-terminal subdomain (C1-C2 and C3-C4) and one bond in C-terminal subdomain (**Figure 3C**). Saposin-like domain is stabilized by the other three disulfide bonds (C5-C10, C6-C9 and C7-C8) (**Figure 3D**). Subgroup I-A proteases are synthesized and transported to vacuoles by the help of saposin-like domain. Once reached the vacuoles, saposin-like domains are cleaved to form mature enzymes (Faro and Gal, 2005).

Subgroup I-B is only found in Brassicaceae, and there are two members in *Arabidopsis*, which are AT4G22050 and AT1G69100. No propeptide and saposin-like domains are found in their sequence, but protease domain organizations are similar to that of I-A proteins (**Figure 3B** shows the alignment between APA1 and AT4G22050, RMSD=0.622). The biological functions and subcellular locations of subgroup I-B are unknown.

### Group II

Group II is distributed in Embryophyta, and 21 *Arabidopsis* genes are classified into this group. When compared to Group I, group II members have no saposin-like domain, and they have 6~7 disulfide bonds in the mature protease formed by 12~14 cysteine residues. Group II was further classified into four subgroups (II-A to II-D).To explore the differences of these four subgroups, four genes are analyzed. APCB1 represents subgroup II-A, APF1(AT2G17760) represents subgroup II-B, AT5G43100 represents subgroup II-C, and A36(AT5G36260) represents subgroup II-D. All subgroups are Embryophyta subgroups.

**Figure 4:**
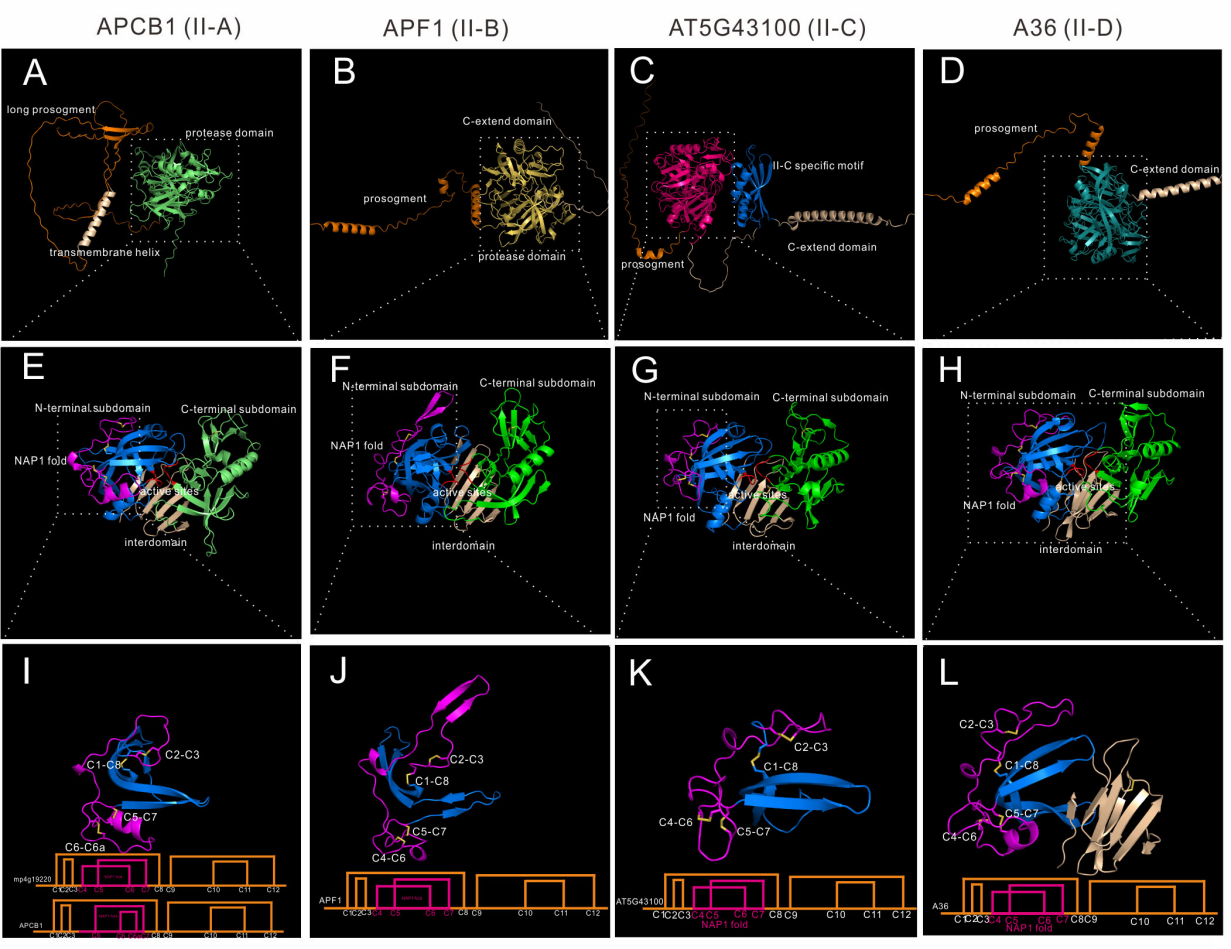
AlphaFold predicted structure models of group II (subgroup II-A to II-D). **(A–D)** Overall structures of APCB1, APF1, AT5G43100 and A36. **(E–H)** Protease domain of APCB1, APF1, AT5G43100 and A36. **(I–L)** NAP1 fold structure and disulfide bond topology of APCB1, APF1, AT5G43100 and A36.

Subgroup II-A has 4 genes in *Arabidopsis*, these four genes were previously identified as nucellin-like type APs (Faro and Gal, 2005). Members of II-A are structural similar (RMSD between 0.541 and 0.919). There is only one homologue in *Marchantia polymorpha*, which is mp4g19220, and APCB1 has the highest similarity to mp4g19220 (RMSD=0.826). To explore the conservation of disulfide bond topology, mp4g19920 cystein residues were numbered as C1-C12 in group II. APCB1 is membrane located protease with a long prosegment (**Figure 4A**). The N-terminal subdomain and C-terminal-subdomain is anchored *via* six stranded interdomain β-sheet (**Figure 4E**). The NAP1 fold of APCB1 is slightly different from mp4g19220, which lacks C4 residue and there is an extra C6a residue (**Figure S7**). Thus, the NAP1 fold of APCB1 is stabilized by C5-C7 and C6-C6a bonds.

Subgroup II-B contains 7 genes in *Arabidopsis*, and 4 of 7 genes are tandem repeated genes (AT5G51330, AT5G51340, AT5G51350 and AT5G51360). Here we take APF1 as an example, which has a prosegment and a C-extend domain (**Figure 4B**).The protease domain of APF1 is similar to APCB1 (**Figure 4F**), except the structure around C2-C3, which is composed of two β-sheet rather than a loop (**Figure 4J**). The disulfide bond topology is the same as mp4g12920. Some members in this subgroup has 14 cysteine residues in its protease domain, for example, an extra pair of disulfide bond exists in C-terminal subdomain in AT5G10080 (C9b-C9c, mp4g12920 number).

Subgroup II-C contains two genes in *Arabidopsis*, which are AT5G43100 and AT3G50050. These two genes have a very long C-extend domain (**Figure 4C**). Based on the AlphaFold predicted structures, these two members have a unique II-C specific motif in the C-terminal extend domain (**Figure 4C**). Though the biological function of II-C specific motif is unknown, AlphaFold predicted structures can be used to unravel previously undetected structures. The protease domain is similar to APCB1 (**Figure 4G**) and has the same disulfide bond topology of mp4g19220 (**Figure 4K**).

Subgroup II-D has 8 genes in *Arabidopsis*, and the best studied members are A36 (AT5G36260) and A39 (AT1G65240) (**Figure 4D**), which are GPI anchored membrane bounded proteases and play vital roles in pollen and ovule development (Gao et al., 2017). The protease domain is similar to other group II members (**Figure 4H**) and has the same disulfide topology of mp4g19220, but the interdomain is slightly different, which is composed of eight β-sheets (**Figure 4L**).

### Group III

Though Group III neither has saposin-like domain or C-extend domain, it is very complex, as shown by the phylogenetic tree. Group III is also the largest group in A1 family. Many genes are specific to one species, as seen in *Marchantia polymorpha* and *Amborella trichopoda*, indicating some group III members may be species specific. Here we classify 38 *Arabidopsis* members as well as most members in other species (except *Marchantia polymorpha* and *Amborella trichopoda* specific subgroups) into seven subgroups (subgroup III-A to III-G). To explore the structure features of these seven subgroups, seven genes are analyzed in detail. AT3G52500 represents III-A, ASPG1/AED2 represents III-B, NANA represents III-C, PCS1 represents III-D, ASPR1 represents III-E, UND represents III-F, and CDR1 represents III-G.

**Figure 5:**
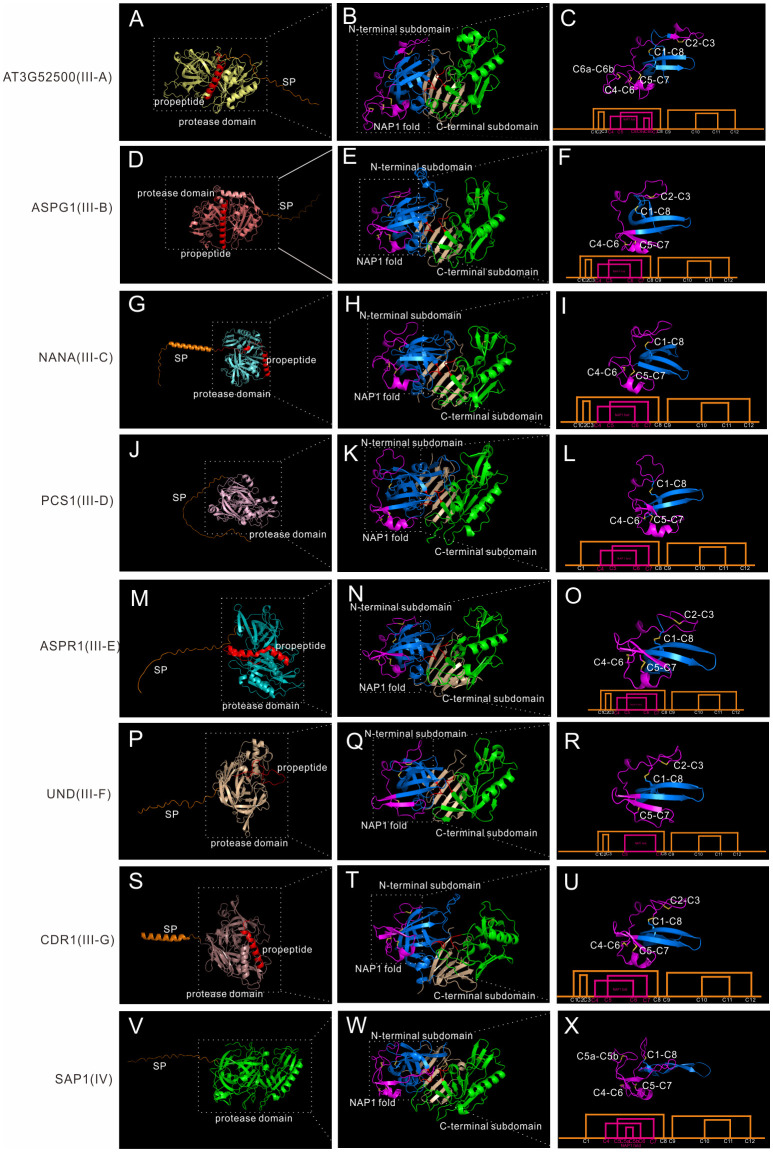
AlphaFold predicted structure models of group III and group IV. **(A-C)** Structure and disulfide bond topology of III-B(AT3G52500); **(D–F)** Structure and disulfide bond topology of III-C (ASPG1); **(G–I)** Structure and disulfide bond topology of III-D (NANA); **(J–L)** Structure and disulfide bond topology of III-E (PCS1); **(M–O)** Structure and disulfide bond topology of III-F (ASPR1); **(P–R)** Structure and disulfide bond topology of III-G (UND); **(S–U)** Structure and disulfide bond topology of III-H (CDR1); **(V–X)**. Structure and disulfide bond topology of IV (SAP1).

III-A members are distributed in Spermatophyta (**Figure S6B**). Subgroup III-A contains 3 genes, which are AT3G52500, AT5G45120 and AT4G16563. This group has 14 cysteine residues, and the NAP1 fold is stabilized by three disulfide bonds. Here we take AT3G52500 as the example, which is composed of a signal peptide, a propeptide and the protease domain (**Figure 5A**). Similarly, the protease is composed of N-terminal subdomain and C-terminal subdomain, and a six stranded interdomain β-sheet (**Figure 5B**). There is an extra disulfide bond between C6 and C7 (**Figure 5C**).

III-B members are distributed in Embryophyta (**Figure S6B**). Subgroup III-B contains 12 genes in *Arabidopsis*, and the best studied member is ASPG1/AED2 (AT3G18490), which plays roles in drought tolerance through ABA signaling and systemic acquired resistance (Yao et al., 2012; Breitenbach et al., 2014). AED1, AED2 and AT5G24820 are also classified into this subgroup. Though only a few group III members have intron(s) in its gene structure, most members are classified into this subgroup (7 out of 8 members in *Arabidopsis*).Here we take ASPG1 as the example, which is composed of a SP, a propeptide and the protease domain (**Figure 5D**). The protease domain is similar to other members (**Figure 5E**). ASPG1 has 12 cysteine residues and the disulfide bond topology is the same as ASPR1 (**Figure 5F**).

III-C members are distributed in Embryophyta (**Figure S6B**). Subgroup III-C contains 4 genes, and the best studied member is NANA (AT3G12700), which is located in the chloroplast (Paparelli et al., 2012). The most remarkable feature of NANA is the unexpected subcellular location of chloroplast, though the chloroplast transit peptide has not been identified (Paparelli et al., 2012). NANA is composed a signal peptide, a propeptide and the protease domain (**Figure 5G**). NANA lacks C2-C3 bond, thus there is only three disulfide bonds in the N-terminal subdomain (**Figures 5H, I**).

III-D members are distributed in Spermatophyta (**Figure S6B**). Subgroup III-D contains 4 genes in *Arabidopsis*, and the best studied member is PCS1 (AT5G02190), which plays a vital role in gametogenesis and embryogenesis PCD (Ge et al., 2005). PCS1 has a long serine-rich signal peptide, which maybe related with its ER subcellular location (**Figure 5J**) (Ge et al., 2005). The overall protease structure is similar to other group III members (**Figure 5K**). PCS1 lacks C2-C3 bond, and the disulfide bond topology is the same as NANA (**Figure 5L**).

III-E members are distributed in Spermatophyta (**Figure S6B**). Subgroup III-E contains only one genes in *Arabidopsis*, namely AT2G03200(ASPR1), which plays role in lateral root development (Soares et al., 2019a). But this group contain 39 genes in *Oryza sativa*, indicating this subgroup is greatly expanded in the monocots. The biochemical characteristics of ASPR1 is similar to CDR1 and ECS1/2, for example, they are active without propetide removal and inhibitor pepsatin cannot totally inhibit their activity (Simões et al., 2007; Soares et al., 2019a; Yu et al., 2021). ASPR1 has a signal peptide, a propeptide and the protease domain (**Figure 5M**). The overall structure is similar, but there is an extra α-helix in the interdomain beta-sheet (**Figure 5N**). ASPR1 has 12 cysteine residues (**Figure 5O**).

III-F members are distributed in Spermatophyta (**Figure S6B**) and contains 5 genes in *Arabidopsis*. The best studied gene is UND (AT4G12920), which plays key roles in tapetal programmed cell death (PCD) activated by transcription factor MYB80 (Phan et al., 2011). UND has a signal peptide, a possible propetide and the protease domain (**Figure 5P**). The overall protease structure is similar to other members (**Figure 5Q**), but UND lacks C4-C6 bond in the NAP1 fold (**Figure 5R**).

III-G members are distributed in Spermatophyta (**Figure S6B**). Subgroup III-G contains 9 genes with similar structures (**Figure S8**), and several genes such as CDR1 (AT5G33340), ECS1 (AT1G31450) and ECS2 (AT2G35615) have been well investigated (Xia et al., 2004; Yu et al., 2021). CDR1 is composed of a secreted SP, a propeptide and the protease domain (**Figure 5S**). And the overall protease structure is similar to all other members (**Figures 5T**; **S8**). The disulfide bond topology is the same as ASPR1 (**Figure 5U**). Some members in this subgroup has variation in disulfide bond topology. In AT2G28010, AT2G28030, AT2G28040, AT2G28220 and AT2G28225, C4 and C5 residues are lacked in NAP1 fold, thus only C6-C7 bond exists in the NAP1 fold, and an extra disulfide bond exists in C-terminal subdomain (C9a-C11a), which may be related to its substrate specificity (**Figures S8B, C**).

### Group IV

Group IV members are distributed in Spermatophyta (**Figure S6B**). In *Arabidopsis*, six genes are classified into group IV, and the best studied members are SAP1 (AT1G03220) and SAP2 (AT1G03230), which play vital roles in antibacterial resistance by cleaving bacterial protein MucD (Wang et al., 2019). Here we take SAP1 as the example, which is composed of a signal peptide and a protease domain (**Figure 5V**).The overall protease structure is similar to group III members (eg.SAP1+ASPR1, RMSD = 1.534), and the protease domain is composed of N-terminal subdomain and C-terminal subdomain anchored *via* six interdomain β-sheet (**Figure 5W**). However, there a notable feature of SAP1, which is its unusual active sites. In contrast to having a canonical active site of DTGS and DSGT, SAP1 has an active site of DLGG and SSVN (**Figure 5B**). The unusual actives sites may explain why this group is frequently ignored or eliminated in previously classifications (**Figures S1**–**S3**) (Beers et al., 2004; Faro and Gal, 2005; Takahashi et al., 2008). The protease activity of SAP1 and SAP2 can be inhibited by aspartic protease inhibitor pepsatin (Wang et al., 2019). To further explore its protease activity, we analyzed the crystal structure of human pepsin and the structure model of SAP1 since both activities can be inhibited by pepsatin (**Figure S9**) (Fujinaga et al., 1995; Wang et al., 2019). The structure of SAP1 is slightly similar to human pepsin (**Figure S7B**, RMSD = 10.989), thus providing insights that these proteins are indeed aspartic protease members. Based on the structure model predicted by AlphaFold, SAP1 has 12 cysteine residues in the protease domain, but the disulfide bond topology is different (**Figure 5C**, ASPR1 number). SAP1 lacks C2-C3 bond, but there are an extra bond between C5 and C6 (C5a-C5b), thus it has a less flexible NAP1 fold (**Figure 5X**).

## Conclusion

In this paper, integrated information of phylogenetic analysis, gene structure analysis, and AlphaFold predicted structures were applied to reclassified A1 aspartic protease family in *Arabidopsis*. We showed that AlphaFold predicted structures can provide valuable insights for the classification of A1 aspartic protease family, especially for those nucellin-like type and atypical type members. Based on these information, previously typical-type was classified into group I, nucellin-like type and atypical type were classified into group II and group III. We also identified a new Spermatophyta distributed group IV without canonical active sites. Group II and group III were further classified into four and seven subgroups respectively. This classification system provides a better resolution in the aspect of gene structure and protein structures, which were not carefully considered in previous classification revision. It should be noted that classification is always a on-going process. The biological functions and biochemical properties of many A1 members are still lacked, further investigations of these members in the future should provide more insights for the classification of this complex but important family.

## Data availability statement

The datasets presented in this study can be found in online repositories. The names of the repository/repositories and accession number(s) can be found in the article/**Supplementary Material**.

## Author contributions

XY conceived and designed the study, XY and YD analyzed the data, and XY,YD and HT wrote the manuscript. All authors contributed to the article and approved the submitted version.

## Supplementary material

Supplementary Figure 1 A1 aspartic protease family classification provided by Beers et al. (Beers et al., 2004). The classification system was drawn based on phylogenetic tree of 70 *Arabidopsis* A1 members,the missing genes are labeled by red star, and boostrap value > 70 are labeled by red circle at the internal nodes.

Supplementary Figure 2 A1 aspartic protease family classification provided by Faro & Gal (Faro and Gal, 2005). The classification system was drawn based on phylogenetic tree of 70 *Arabidopsis* A1 members,the missing genes are labeled by red star, and boostrap value > 70 are labeled by red circle at the internal nodes.

Supplementary Figure 3 A1 aspartic protease family classification provided by Takahashi et al., (Takahashi et al., 2008). The classification system was drawn based on phylogenetic tree of 70 *Arabidopsis* A1 members,the missing genes are labeled by red star, and boostrap value > 70 are labeled by red circle at the internal nodes.

Supplementary Figure 4 AlphaFold predicted structure models of AT3G30770 and AT3G29750.

Supplementary Figure 5 Phylogenetic tree of 70 *Arabidopsis* A1 aspartic protease family. Four groups (Group I-III) are classified.

Supplementary Figure 6 Average exon numbers in each subgroup and gene numbers in 12 selected species. **(A)** Average exon numbers in each subgroup, scale bar indicates mean with SD, n indicates gene numbers in each subgroup; **(B)** gene numbers of each subgroup in 12 selected species, MP indicates *Marchantia polymorpha* specific members, ATR indicates *Amborella trichopoda* specific members.

Supplementary Figure 7 Multiple sequence alignment of APCB1 and its homologues. Two Arabidopsis sequences (APCB1 and AT4G33490), one rice homologue (Os02g49800), one Amborella trichopoda homologue (ATRAPCB1), and one Marchantia polymorpha homologue (mp4g19220) were aligned. Cystein residues (C1-C8,mp4g19220) were colored by yellow. APCB1 lacks C4 residue and has an extra C6a residue.

Supplementary Figure 8 Structure alignment of subgroup III-G members. **(A)** Structure alignment of CDR1,ECS1 and AT2G28010,RMSD are shown; **(B)** Disulfide bond topology between CDR1 and AT2G28010 are shown, red indicates CDR1, and yellow indicates AT2G28010; **(C)** Disulfide bond topology schematic figure of CDR1 and AT2G28010.

Supplementary Figure 9 Structure alignment of human pepsin and SAP1. **(A)** Structure of human pepsin with inhibitor pepsatin, pepsatin was showed by red color; **(B)** Structure alignment of human pepsin (cyan) and SAP1(green), RMSD = 10.989.

Supplementary Table 1
*Arabidopsis* A1 aspartic protease family members searched by PF00026.

Supplementary Table 2
*Arabidopsis* A1 aspartic protease family members searched by the custom made HMM profile.

Supplementary Table 3 Eliminated genes in Arabidopsis.

Supplementary Table 4 Genome information of 12 selected species.

Supplementary Table 5 Average intron numbers of group I-IV in 12 selected species.

Supplementary Table 6 Detailed gene structure information of 1200 genes in 12 selected species.
