# Supplementary figures and images for: Phylogenetic and AlphaFold predicted structure analyses provide insights for A1 aspartic protease family classification in Arabidopsis
Source: Front Plant Sci. 2023 Feb 3;14:1072168. doi: 10.3389/fpls.2023.1072168 (PMC9937552; doi:10.3389/fpls.2023.1072168)

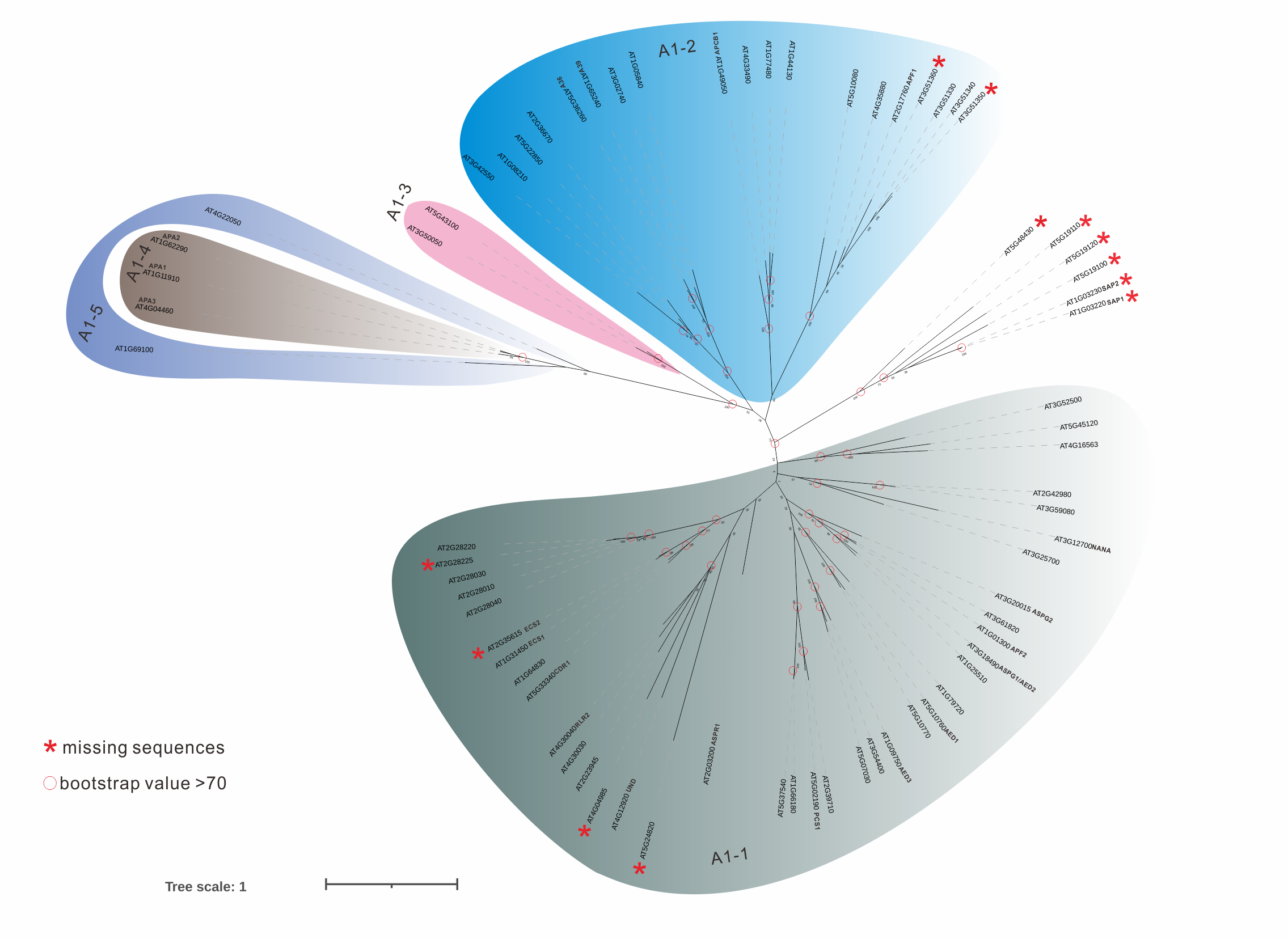

Supplement: Supplementary Figure 1 — A1 aspartic protease family classification provided by Beers et al. (Beers et al., 2004). The classification system was drawn based on phylogenetic tree of 70 Arabidopsis A1 members,the missing genes are labeled by red star, and boostrap value > 70 are labeled by red circle at the internal nodes. [file Image_1.tif]

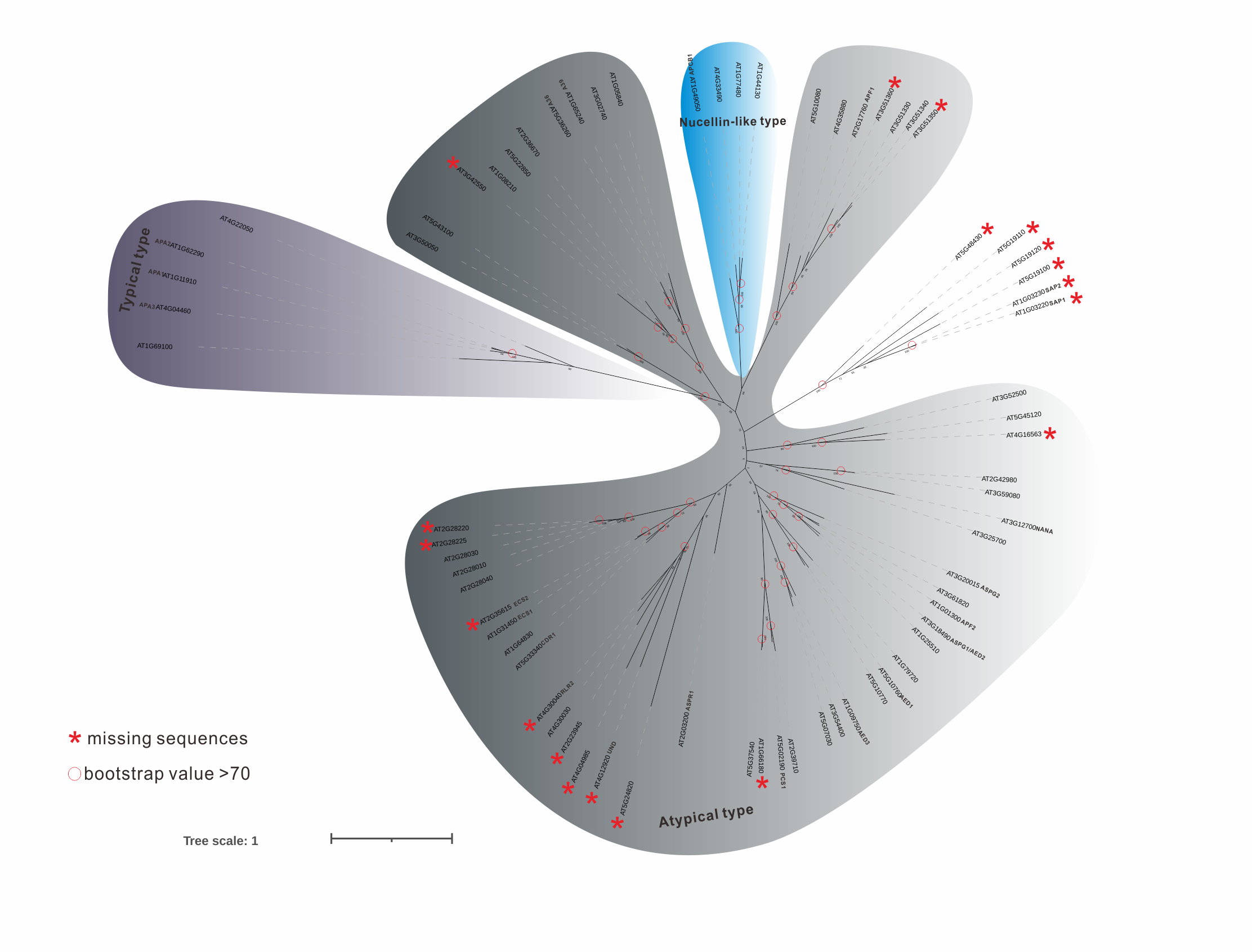

Supplement: Supplementary Figure 2 — A1 aspartic protease family classification provided by Faro & Gal (Faro and Gal, 2005). The classification system was drawn based on phylogenetic tree of 70 Arabidopsis A1 members,the missing genes are labeled by red star, and boostrap value > 70 are labeled by red circle at the internal nodes. [file Image_2.tif]

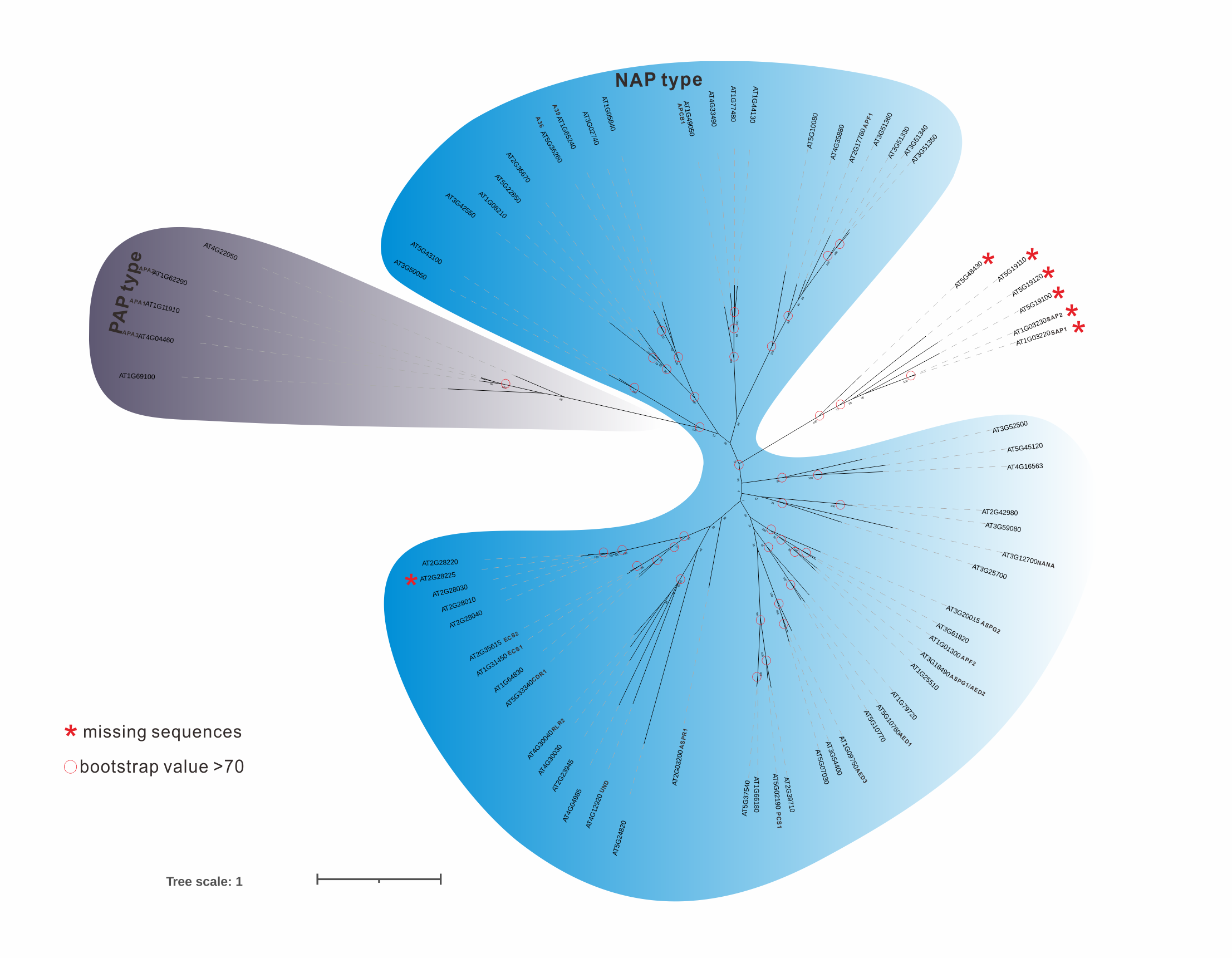

Supplement: Supplementary Figure 3 — A1 aspartic protease family classification provided by Takahashi et al., (Takahashi et al., 2008). The classification system was drawn based on phylogenetic tree of 70 Arabidopsis A1 members,the missing genes are labeled by red star, and boostrap value > 70 are labeled by red circle at the internal nodes. [file Image_3.tif]

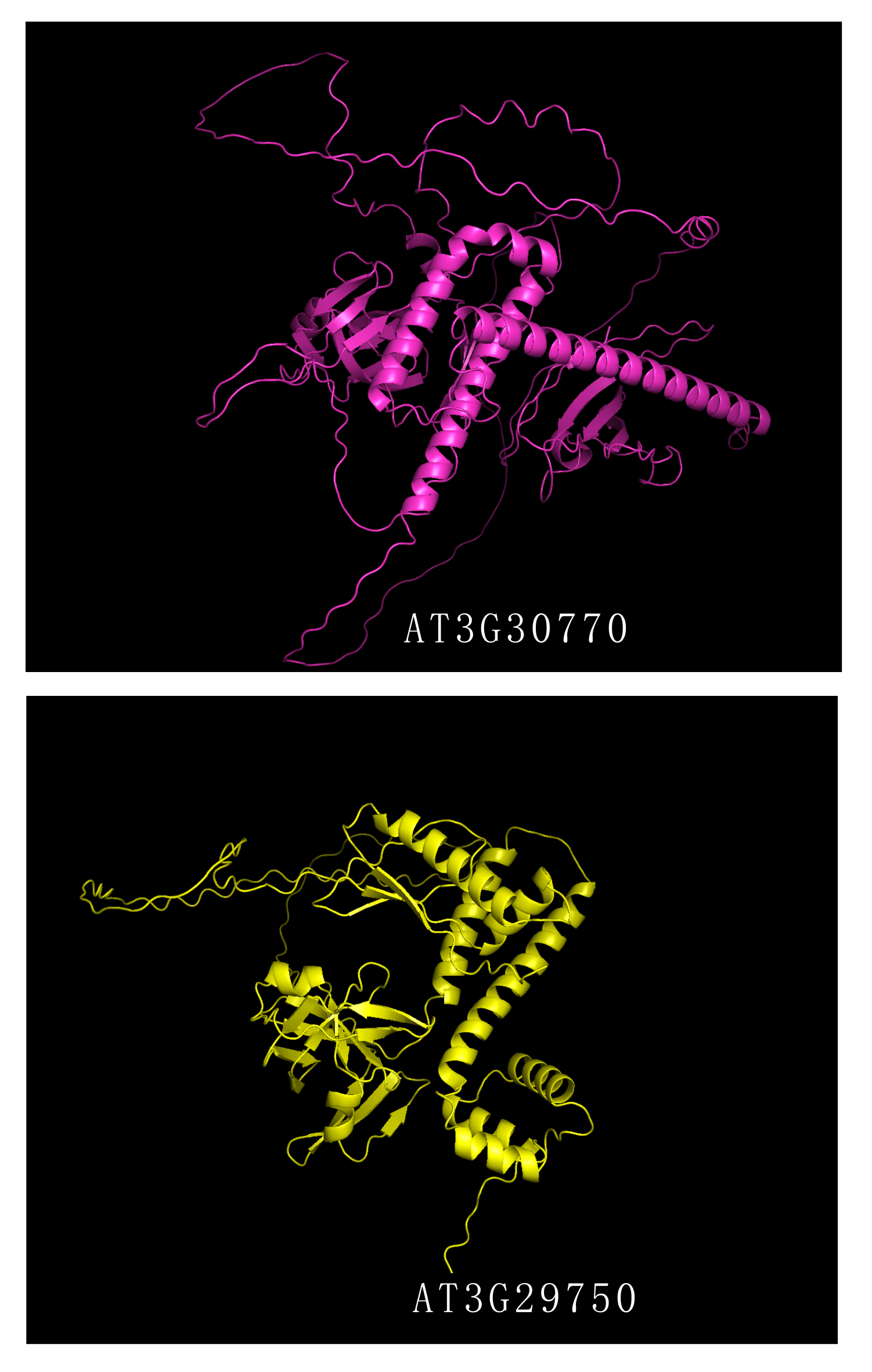

Supplement: Supplementary Figure 4 — AlphaFold predicted structure models of AT3G30770 and AT3G29750. [file Image_4.tif]

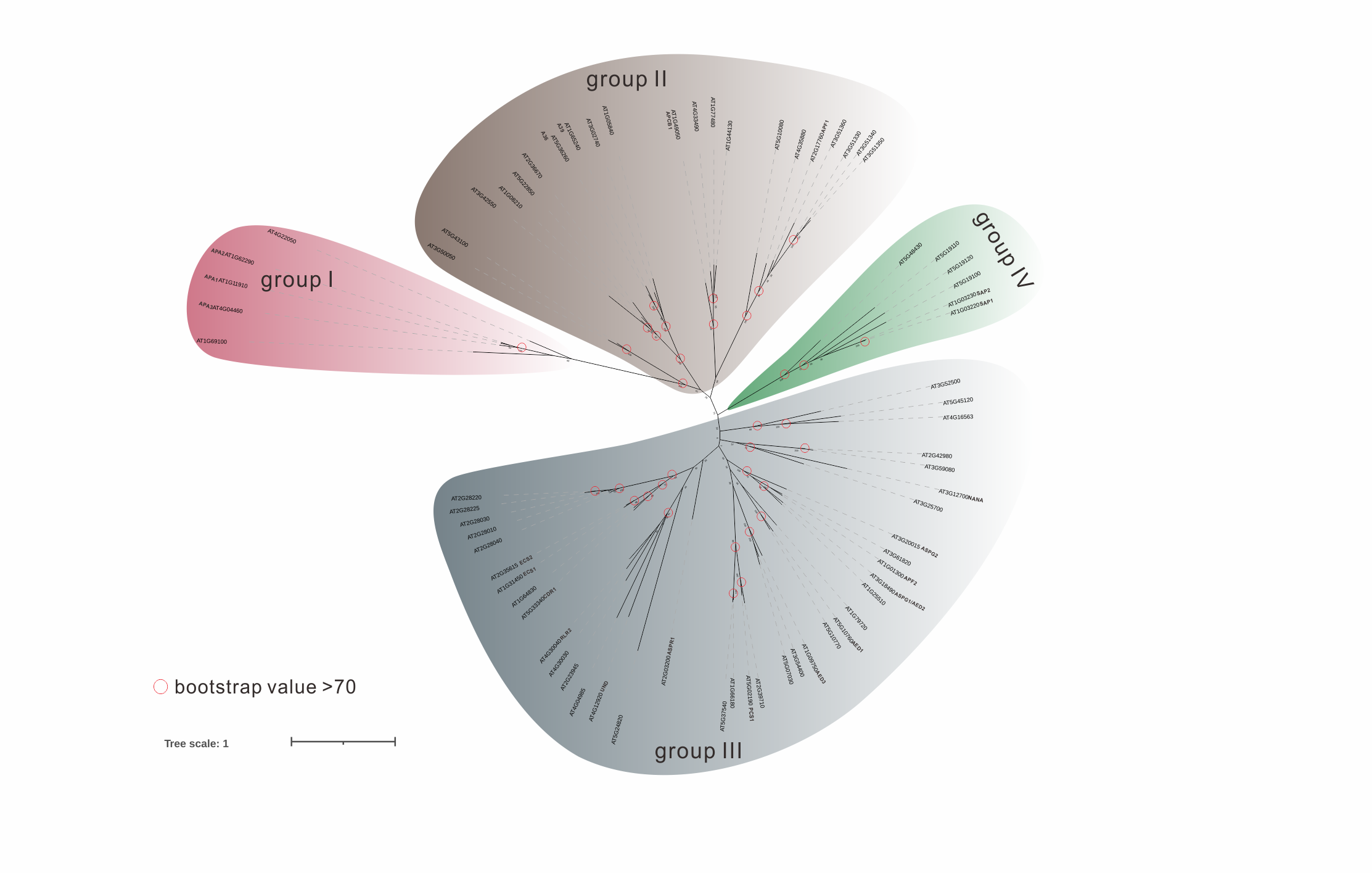

Supplement: Supplementary Figure 5 — Phylogenetic tree of 70 Arabidopsis A1 aspartic protease family. Four groups (Group I-III) are classified. [file Image_5.tif]

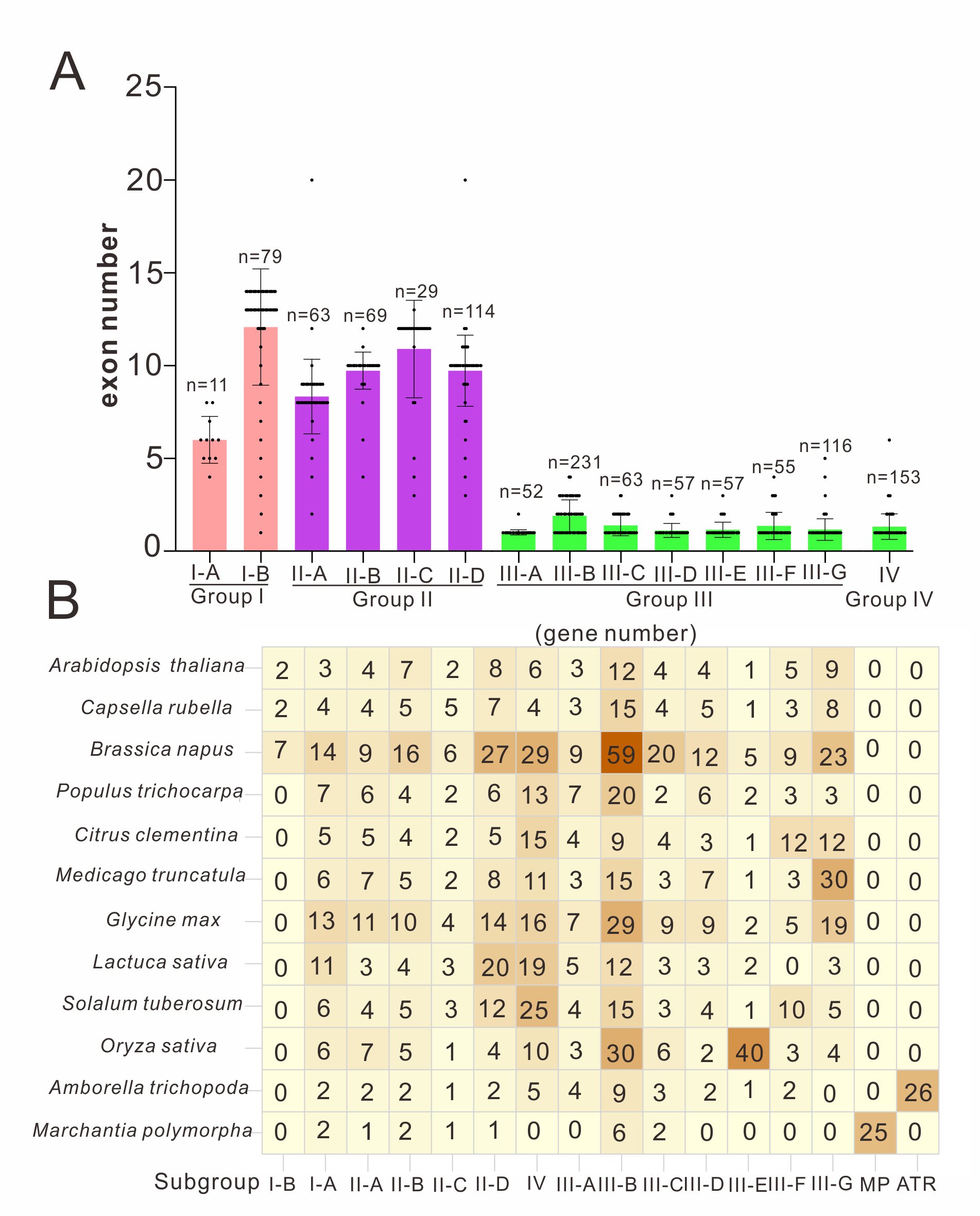

Supplement: Supplementary Figure 6 — Average exon numbers in each subgroup and gene numbers in 12 selected species. (A) Average exon numbers in each subgroup, scale bar indicates mean with SD, n indicates gene numbers in each subgroup; (B) gene numbers of each subgroup in 12 selected species, MP indicates Marchantia polymorpha specific members, ATR indicates Amborella trichopoda specific members. [file Image_6.tif]

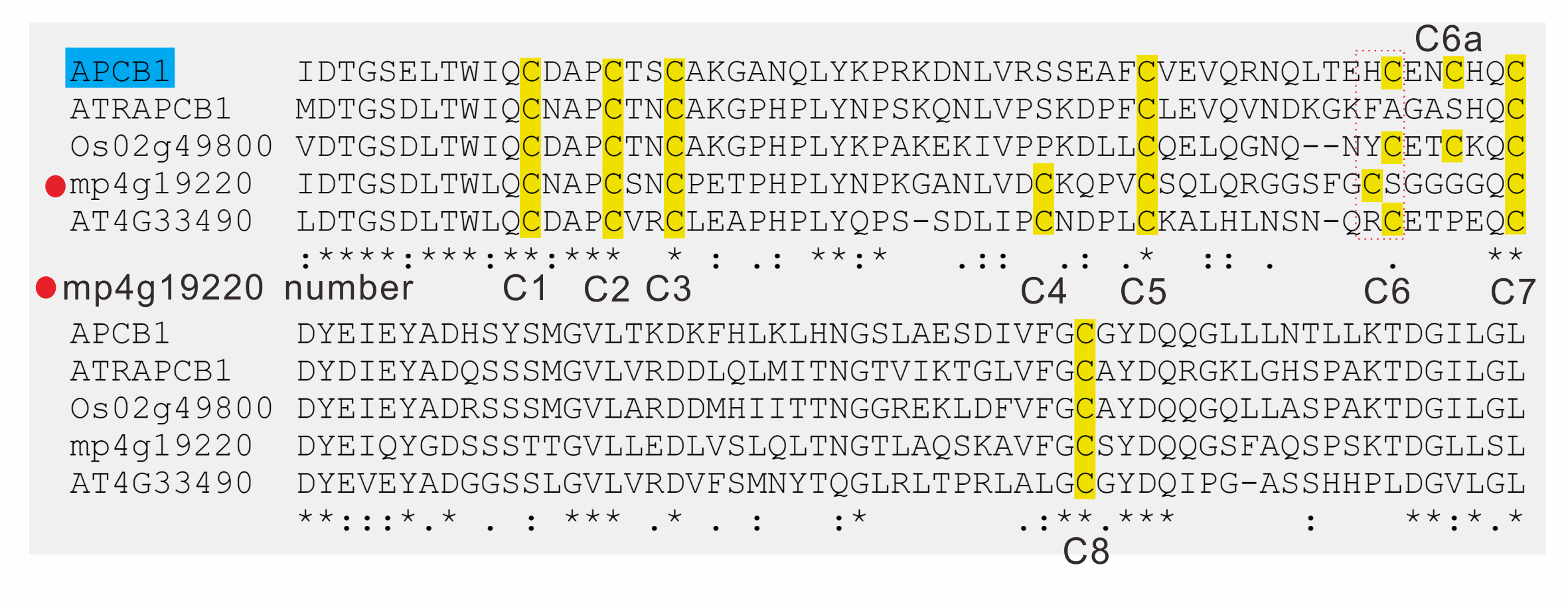

Supplement: Supplementary Figure 7 — Multiple sequence alignment of APCB1 and its homologues. Two Arabidopsis sequences (APCB1 and AT4G33490), one rice homologue (Os02g49800), one Amborella trichopoda homologue (ATRAPCB1), and one Marchantia polymorpha homologue (mp4g19220) were aligned. Cystein residues (C1-C8,mp4g19220) were colored by yellow. APCB1 lacks C4 residue and has an extra C6a residue. [file Image_7.tif]

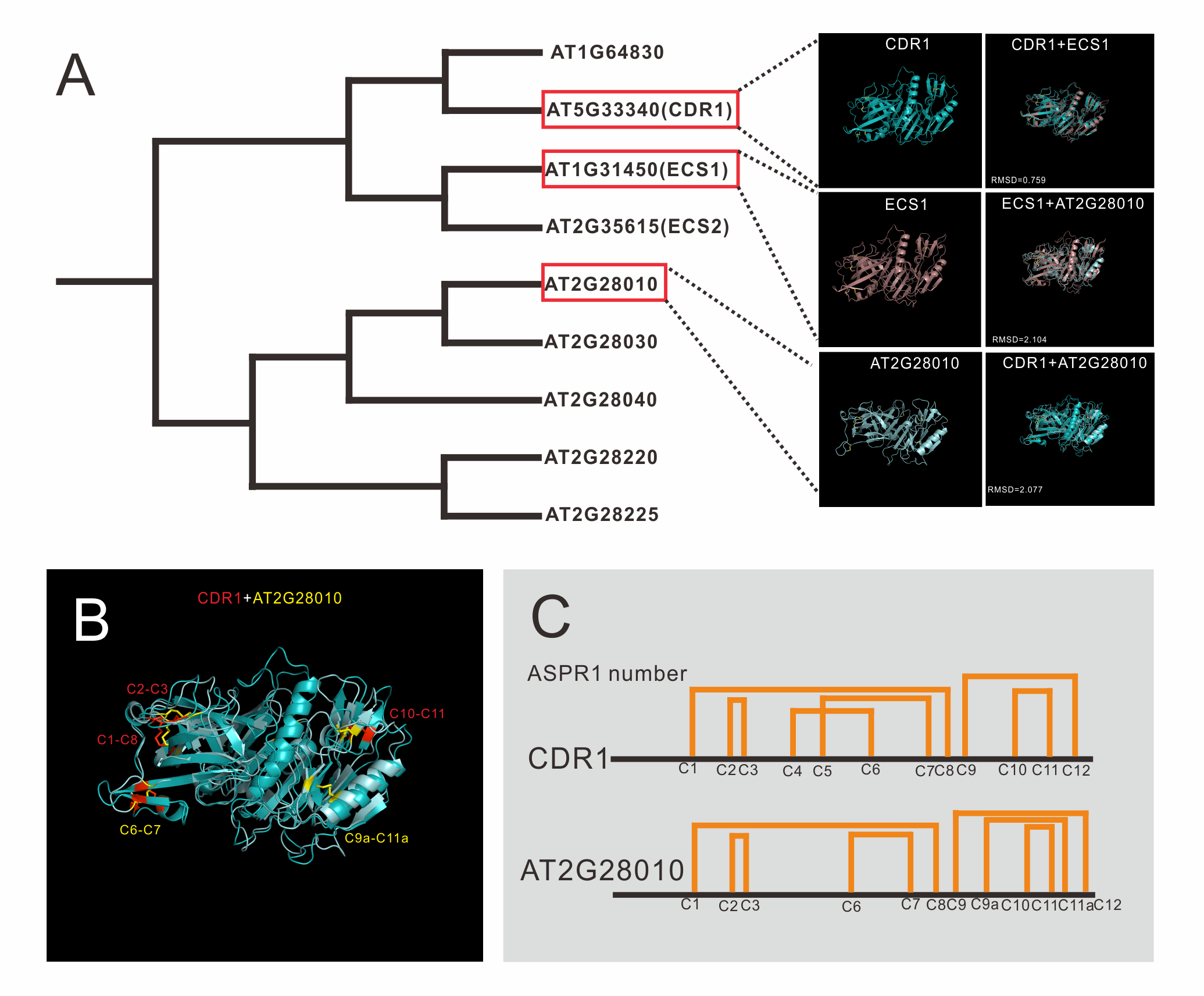

Supplement: Supplementary Figure 8 — Structure alignment of subgroup III-G members. (A) Structure alignment of CDR1,ECS1 and AT2G28010,RMSD are shown; (B) Disulfide bond topology between CDR1 and AT2G28010 are shown, red indicates CDR1, and yellow indicates AT2G28010; (C) Disulfide bond topology schematic figure of CDR1 and AT2G28010. [file Image_8.tif]

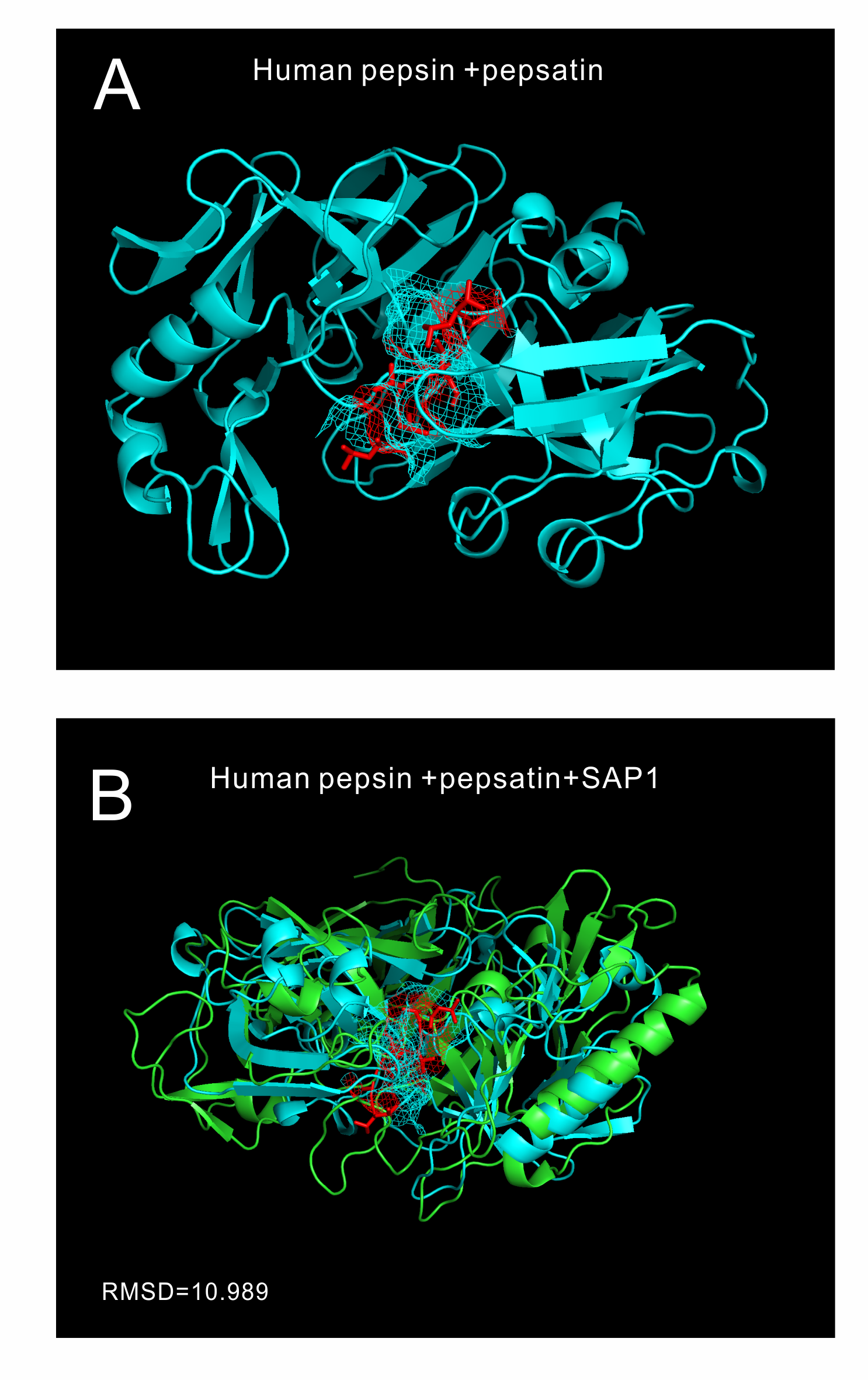

Supplement: Supplementary Figure 9 — Structure alignment of human pepsin and SAP1. (A) Structure of human pepsin with inhibitor pepsatin, pepsatin was showed by red color; (B) Structure alignment of human pepsin (cyan) and SAP1(green), RMSD = 10.989. [file Image_9.tif]
