# Supplementary material for: Phylogenetic and AlphaFold predicted structure analyses provide insights for A1 aspartic protease family classification in Arabidopsis
Source: Front Plant Sci. 2023 Feb 3;14:1072168. doi: 10.3389/fpls.2023.1072168 (PMC9937552; doi:10.3389/fpls.2023.1072168)

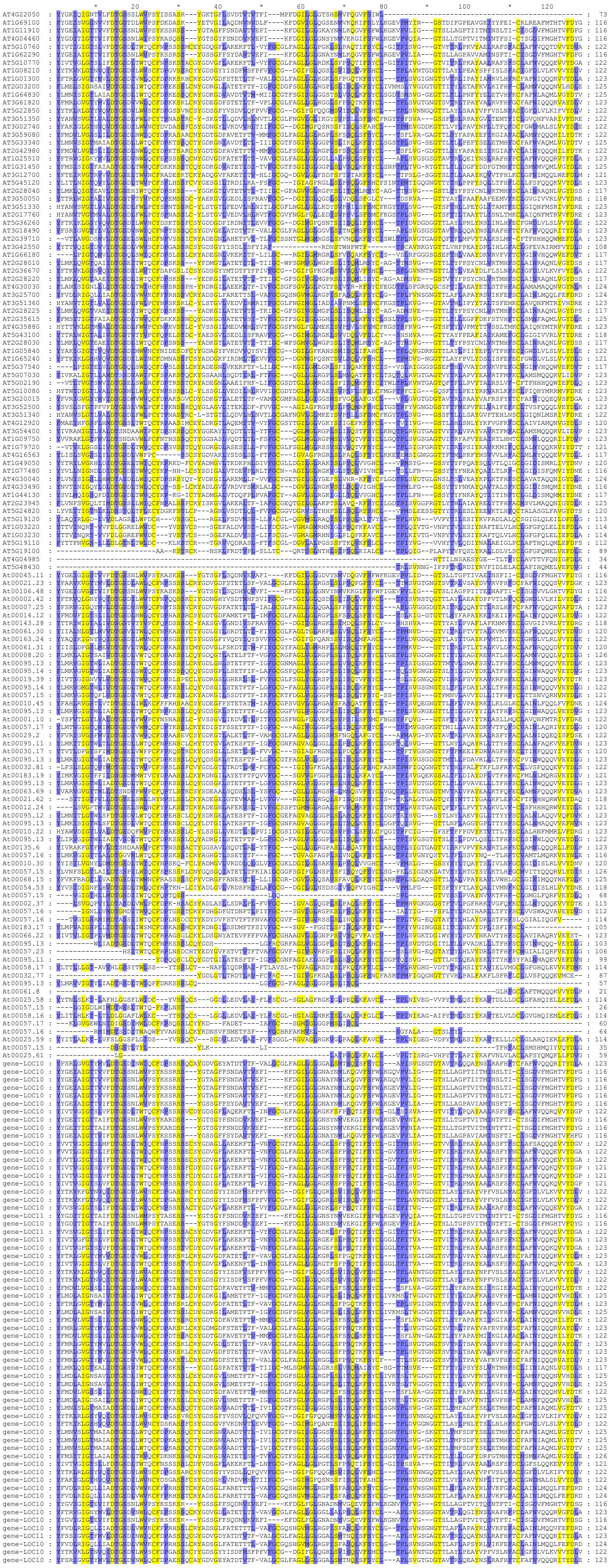

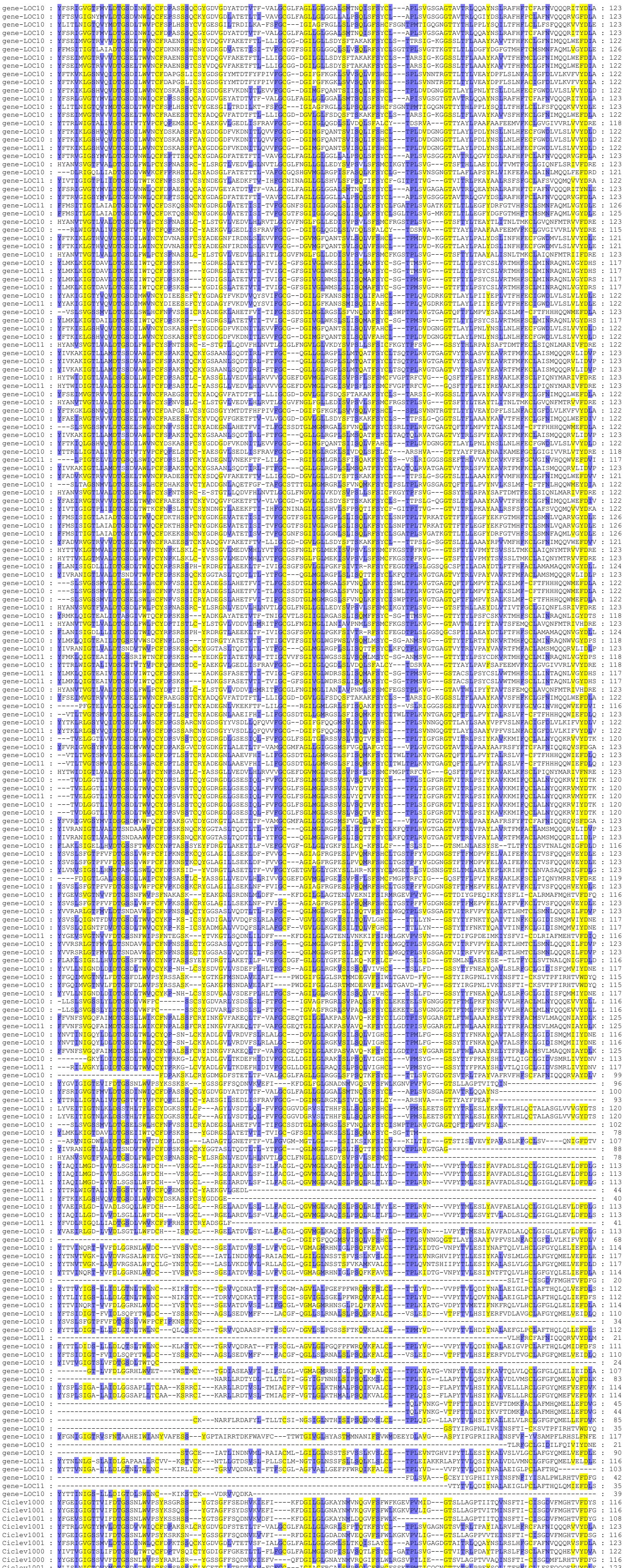

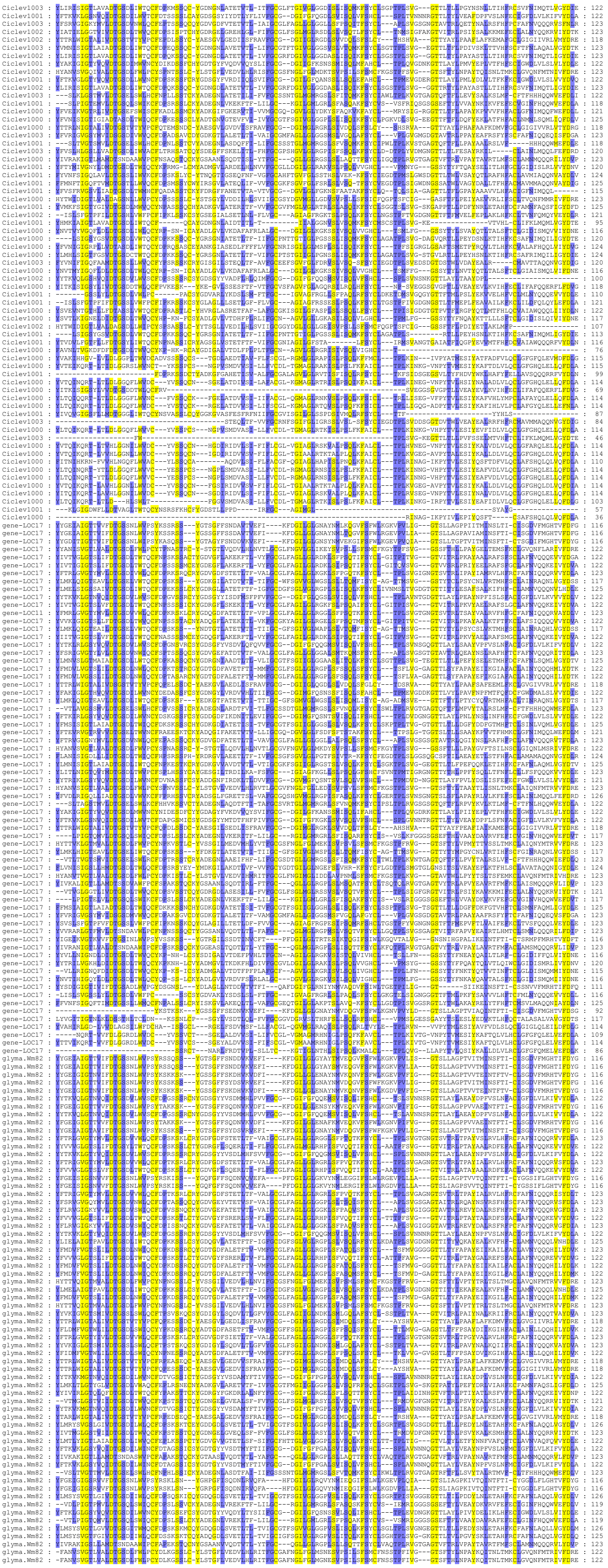

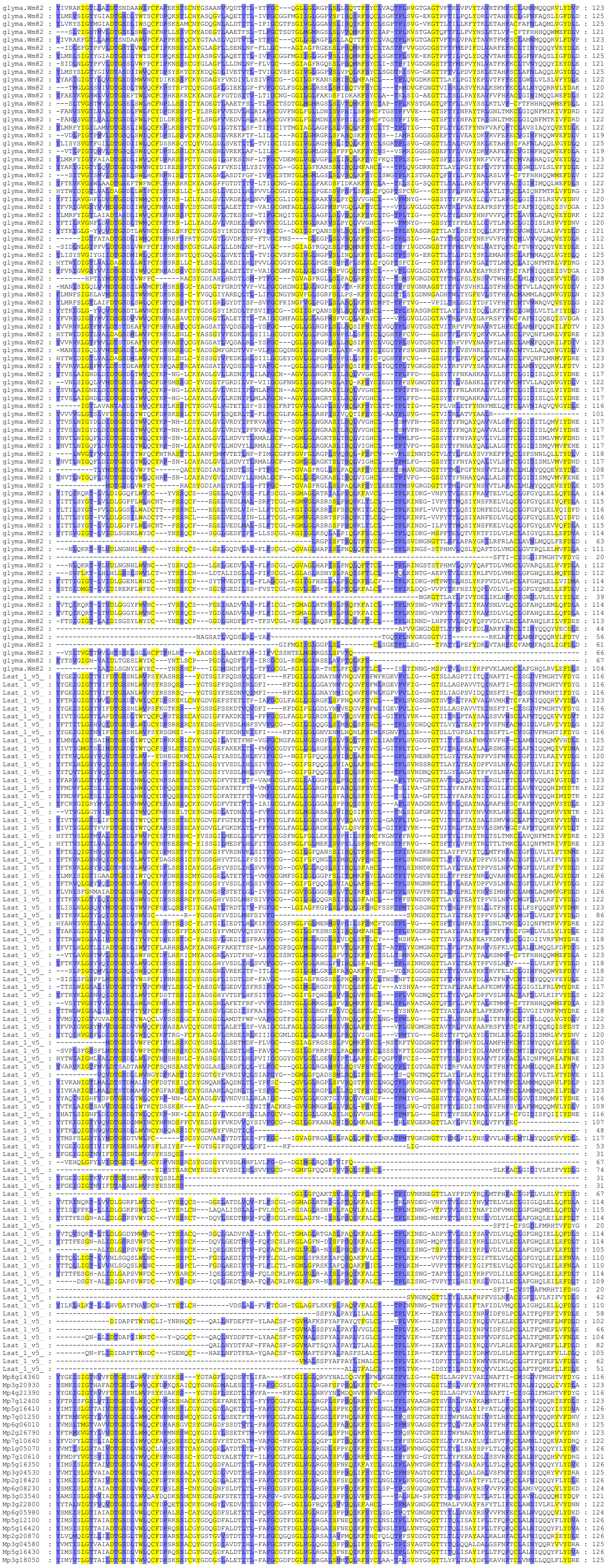

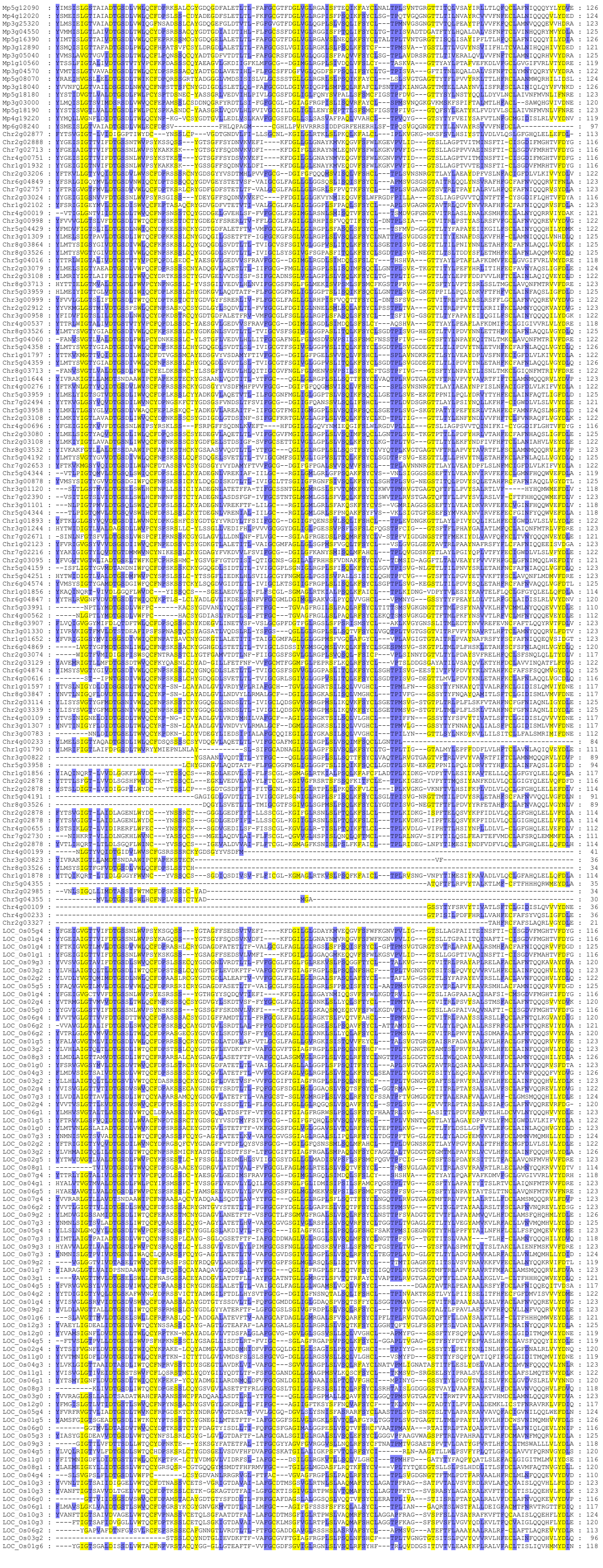

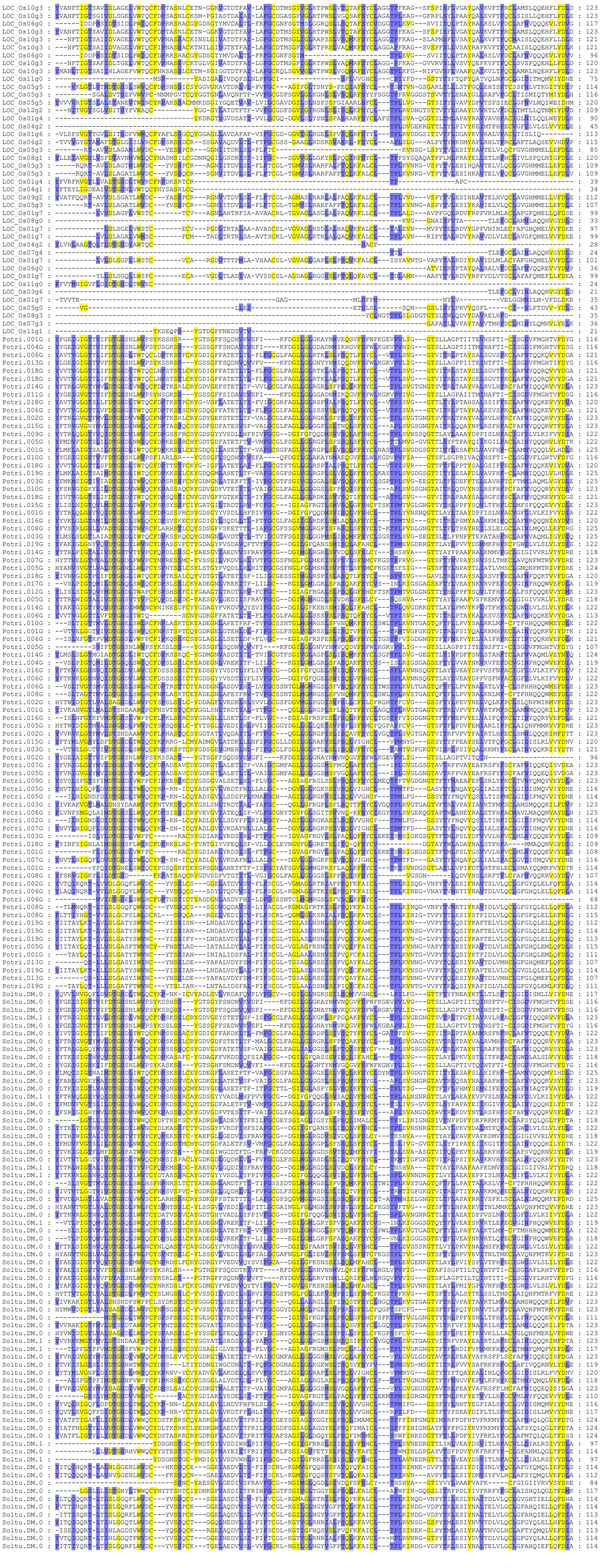

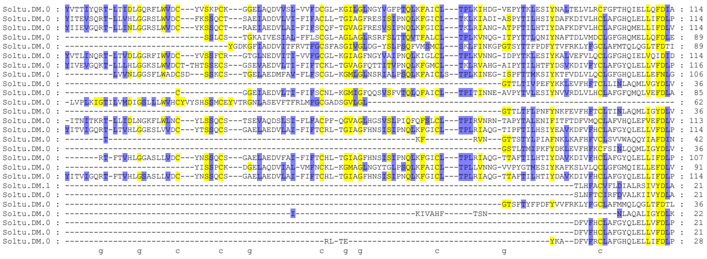

Supplement: Supplementary Table 4 — Genome information of 12 selected species. [file DataSheet_1.pdf]
